# Protein phosphatase 4 regulates apoptosis in leukemic and primary human T-cells

**DOI:** 10.1016/j.leukres.2009.05.013

**Published:** 2009-11

**Authors:** Mirna Mourtada-Maarabouni, Gwyn T. Williams

**Affiliations:** Institute for Science and Technology in Medicine and School of Life Sciences, Huxley Building, Keele University, Keele ST5 5BG, UK

**Keywords:** Leukemia, T-cells, Apoptosis, Kinases/phosphatases, Signal transduction, PEA-15, Mutation, PPX, PPP4

## Abstract

The control of T-cell survival is of overwhelming importance for preventing leukemia and lymphoma. The present report demonstrates that the serine/threonine protein phosphatase PP4 regulates the survival of both leukemic T-cells and untransformed human peripheral blood T-cells, particularly after treatment with anti-leukemic drugs and other cytotoxic stimuli. PP4-induced apoptosis is mediated, at least in part, through de-phosphorylation of apoptosis regulator PEA-15, previously implicated in the control of leukemic cell survival. PP4 activity significantly affects the mutation rate in leukemic T-cells, indicating that PP4 dysfunction may be important in the development and progression of leukemia.

## 1 Introduction

Apoptosis plays a crucial role in regulating homoeostasis in the immune system. This physiological process of cell suicide is essential for T-cell development, for the shaping of the immune repertoire and for coordinating the events leading to immune responses [1–3]. Since the inappropriate lymphocyte survival that results from apoptosis failure is of great significance for the development of leukemia [1–5], dissection of the molecular mechanisms involved in apoptosis is central to understanding leukemogenesis [1,2]. In addition, many anti-leukemic drugs act through induction of apoptosis [1,2,6], so that failure of apoptosis contributes significantly to the appearance of therapy-resistant leukemic cells, particularly during relapse.

Like the cell cycle, apoptosis is regulated by conserved signal transduction pathways that include many specialised activators, effectors and inhibitors [3,6–8]. Genetic or functional alterations of these proteins may lead to inappropriate responses to the microenvironment, ultimately leading to cancer or autoimmunity, on the one hand, or to immunodeficiency, on the other hand [1,2,4,5,9]. One of the most important mechanisms by which both apoptosis and cell proliferation are regulated is the reversible phosphorylation of many proteins which affects their functions in diverse ways. These include increasing or decreasing enzyme activity, e.g. histone deacetylase 7 (HDAC7) [10], translocation of proteins from one cellular compartment to another, e.g. K-Ras [11], marking a protein for degradation, e.g. IκB (Inhibitor of NFκB) [12], or promoting or inhibiting the interaction of one protein with another, e.g. BAD (Bcl-2/Bcl-X associated death promoter) [13]. In particular, the phosphorylation status of many pro-apoptotic (BAD, Bid and Bik) and anti-apoptotic (Bcl-2 and Bcl-X_L_) Bcl-2-family proteins regulates their cellular activity, and consequently controls cell survival [13–16]. The well-balanced opposing operation of protein kinases and phosphatases is therefore critical for the control of cell death and survival. Many oncogenes encode protein kinases and changes in their activity certainly contribute to the process of tumorigenic transformation [17,18]. On the other hand, the exact roles played by protein phosphatases are much less clear, although, logically, they must be of great significance in leukemia progression through their regulation of the phosphorylation status of proteins involved in key survival and proliferation pathways. Indeed, the study of protein phosphatases and their regulation has recently become an expanding field of research, with these protein families becoming recognised as potential therapeutic targets [19,20].

The mammalian serine/threonine phosphatase (PPP) family consists of the most abundant protein phosphatases PP1, Ca^2+^-dependent PP2B, Mg^2+^-dependent PP2c, PP2 (formerly PP2A) and PP2-like phosphatases such as PP6, PP5, PP7 and PP4 [21]. Like other members of the PPP families, the ubiquitously expressed PP4 (PPP4/PPX) exists as a holoenzyme composed of a highly conserved catalytic subunit (PP4c), a structural (A) subunit and regulatory (B) subunits [22]. PP4c shares 65% amino acid identity with both isoforms of PP2 catalytic subunits (PP2cα and PP2cβ), and associates with regulatory subunits which are mostly distinct from PP2 regulatory subunits [23,24]. In addition to the previously identified core regulatory subunits R1 and R2, several other related variable regulatory subunits of PP4 have been identified; R3α, R3β, the eight subunit ATP-dependent chaperonin complex TRiC (also known as CCT) and the α4 regulatory subunit, which is the only subunit shared with PP2 [24–28]. It has now been recognised that PP4 regulates a variety of cellular functions independently of PP2 and it is believed that the interaction of these different regulatory subunits with the catalytic subunit PP4c is central to controlling the functional effects of this enzyme [22].

PP4 complexes are involved in organelle assembly, regulation of microtubule growth, cell migration, and centrosome maturation in mitosis and meiosis as well as spliceosomal assembly via interaction with the SMN complex [22,24,29,30]. Recent studies have shown that PP4c regulates cyclin-dependent kinase 1 (Cdk1) activity and microtubule organization via NDEL1 (nuclear distribution element 1) dephosphorylation [31]. PP4 also regulates several signalling pathways, including NFκB [32,33] and the DNA damage response [34]. PP4 has been reported to regulate haematopoietic progenitor kinase 1 (HPK1) activity in a T-cell receptor (TCR)-dependent manner, suggesting a role for PP4 in T-cell signalling [38]. In addition, PP4 interacts with and down-regulates insulin receptor substrate 4 (IRS4) following tumor necrosis factor alpha (TNFα) stimulation [35], is involved in TNFα-induced activation of the Jun N-terminal protein kinase (Jnk) [36] and has been reported to associate with HDAC3 and to inhibit its activity [37]. Most recently, Nakada et al. [39] have reported that depletion of PP4c results in a prolonged checkpoint arrest in human cells, suggesting that PP4c plays a critical role in dephosphorylating γH2AX after DNA damage.

Emerging evidence indicates that PP4 may play important and complex roles in apoptosis and cell proliferation. Over-expression of PP4c results in an increase in cell death and a decrease in cell proliferation in mouse thymoma cells [40] and in the human embryonic kidney cell line HEK 293T [41]. The present study demonstrates that down regulation of PP4c inhibits apoptosis, increases the proliferation rate, and has a strong influence on gene mutation rate, which is crucial to oncogenesis. On the other hand, PP4 appears to be essential for survival and cell proliferation because of its role in centrosome maturation [29,42]. The observation that PP4 exerts inhibitory, as well as stimulatory, effects on cell growth in some situations is likely to be explained by the activity of different PP4 complexes with distinct subcellular locations and diverse substrates. These observations indicate that a certain level of PP4c activity is likely to be required for cell survival so that deletion of PP4, as well as excessive PP4, can be lethal to cells.

Many studies have also implicated PP2 in the regulation of T-cell signalling, activation and survival [43,44]. Proteomic analysis has shown that changes in PP4 expression levels affect the phosphorylation status of many proteins involved in apoptosis and cell proliferation, including the critical apoptosis regulators BAD and phosphoprotein enriched in astrocytes 15 kD (PEA-15), which were both significantly over-phosphorylated when PP4 was suppressed [41]. PEA-15 is a member of the death effector domain (DED) protein family known to control cell survival [45,46], and specific down-regulation of this protein sensitizes B-cell chronic lymphocytic leukemia cells to TRAIL-induced apoptosis [47]. The analysis reported in the current paper is consistent with direct interaction between PP4c and PEA-15, but not between PEA-15 and PP2.

Since previous experiments with mouse thymoma cells revealed a pro-apoptotic role for PP4c [40], we have extended the analysis of the effects of modulation of PP4c expression on apoptosis and cell proliferation to the human leukemic T-cell lines Jurkat and CEM-C7 and to primary human lymphocytes. These studies reveal the importance of PP4 expression levels in determining the sensitivity of both leukemic and untransformed human lymphocytes to a range of anti-leukemic drugs and other cytotoxic stimuli.

## 2 Materials and methods

### 2.1 Materials

Cisplatin (155663-27-1), butyrate (B5887), okadaic acid (#04511) and dexamethasone (D4902) were purchased from Sigma.

### 2.2 Cell culture

The cloned human T-leukemic cell lines CEM-C7.CMK1 and Jurkat.JKM1 [48] were maintained in RPMI-1640 medium (Sigma) supplemented with 10% heat inactivated fetal calf serum (Hyclone), 2 mM l-glutamine and 200 μg/ml gentamycin (Sigma), at 37 °C in a 5% CO_2_ humidified incubator. All experiments were carried out using cells in logarithmic growth phase.

### 2.3 Primary lymphocyte isolation and culture

Whole blood was collected from healthy volunteers into heparinized tubes. Ethical approval was obtained from the Local Ethical Committee and peripheral blood lymphocytes were prepared and PHA-stimulated as described previously [49].

### 2.4 Determination of cell viability and detection of apoptosis

Cell viability was determined by nigrosin exclusion analysis and by the MTS assay, (Promega; # G5421). The CaspaTag Caspase Activity Kit (Intergen; # S7300-025) was used to detect active caspases in the cells as a marker for caspase-dependent apoptosis. Detection was performed using a fluorescence microscope.

### 2.5 Plasmid DNA transfection

pcDNA3.1-PP4c expression constructs, or the vector alone, were introduced into human leukemic T-cells by electroporation (20 μg DNA at 248 V (CEM-C7) and 293 V (Jurkat), 1050 μF in 0.4 cm cuvettes (Biorad) at room temperature). Efficiency of transfection was 60–70% for CEM-C7 and Jurkat. In order to generate stable cell lines, at 24 h post-transfection cells were cloned in soft agar in Iscoves's medium containing 2 mM glutamine, 20% heat inactivated fetal bovine serum and 0.5 mg/ml G418 (Sigma) for 2–3 weeks at 37 °C in a humidified incubator with 5% CO_2_. Individual colonies were picked and expanded in medium containing 0.5 mg/ml G418 and the level of expression of the gene transfected was monitored by Western analysis or qRT-PCR.

### 2.6 Transfection of primary lymphocytes

Transfection of plasmid DNA and siRNA into primary lymphocytes was carried out by nucleofection (Amaxa Biosystems). Primary lymphocytes were cultured for 5–6 days in the presence of 2.5 μg/ml PHA [50] and transfection was carried out using the Nucleofector kit for stimulated human T-cells (Program T-23; Amaxa # VPA-1002), following the manufacturer's instructions. Transfection efficiency was 60–70%.

### 2.7 Preparation of cells for cell cycle analysis

Preparation of cells and nuclear propidium iodide (PI) staining for cell cycle analysis was performed according to standard procedures, as described previously [49].

### 2.8 Ki-67 labelling

10^6^ cells were supended in 70% ethanol/30% PBS by vortexing and incubated at −20 °C for 2 h. Fixed cells were then washed twice with staining buffer (PBS with 1% fetal calf serum, 0.09% NaN_3_), centrifuged for 10 min at 200 × *g* and they were resuspended in 100 μl in the staining buffer. Ki-67 antibody (Santa Cruz Biotech.; Cat # sc-23900) was added at 1:50 dilution. Cells were incubated for 30 min in the dark. After that time, cells were washed twice with the staining buffer, centrifuged at 200 × *g* for 10 min, resuspended in 100 μl of staining buffer and the secondary antibody (Santa Cruz; Anti-mouse IgG-FITC Cat # sc-2010) added at a dilution of 1:200. Cells were incubated in the dark for 1 h before they were washed twice in staining buffer. After centrifugation, the cells were resuspended in 50 μl of staining buffer and Ki-67 positive cells were detected using a fluorescence microscope.

### 2.9 Clonogenic assay

Long-term survival and proliferation of cells transfected with PP4c expression constructs or with PP4 siRNAs was assessed by the ability of the cells to form colonies in soft agar. An equal proportion of the culture from each experimental condition was diluted in 5 ml Iscove's medium (Sigma) containing 20% heat inactivated fetal calf serum, 10% CEM-C7- or Jurkat-conditioned medium and 0.3% Noble agar (Difco) and plated in 60 mm dishes. Dishes were also overlaid with 2.5 ml Iscove's complete medium containing 10% cell-conditioned medium. The number of colonies formed was counted following 2–3 weeks incubation at 37 °C in 5% CO_2_.

### 2.10 RNA interference

Two different PP4c siRNAs (siRNAs ID# 105835 (PP4s1); siRNA ID# 105834 (PP4s2)), two PP2cβ siRNAs (PP2s1 ID# 5230 and PP2s2 ID# 105844), three different PEA-15 siRNAs (PEA-15s1 ID # 137203; PEA-15s2 ID # 137202; PEA-15s3 ID# 43349), negative control siRNA (siRNA Cat # 4605) and GAPDH (Cat # 4605) gene specific pre-designed siRNA were purchased from Ambion (HPLC purified, annealed and ready to use). To monitor transfection efficiency (70–80% at 48 h), siRNA duplexes were labelled with Cy3 using the *Silencer*™ siRNA labelling kit (Ambion; Cat# 1632), following the manufacturer's instructions. On the day before transfection, cells were sub-cultured in RPMI supplemented with 10% FCS. On the day of transfection, 10^6^ cells (CEM-C7, Jurkat or primary lymphocytes) were centrifuged, washed once in Optimem 1 (Invitrogen; # 51985-026) and resuspended in 400 μl Optimem 1. Cells were incubated with 20 or 100 nM siRNA duplex for 10 min at room temperature in a 0.4 cm gap electroporation cuvette and electroporated for 25 ms at 248 V (CEM-C7) or 293 V (Jurkat and primary lymphocytes) and 1050 μF using a Biorad Gene Pulser. Following electroporation, cells were incubated at room temperature for 20 min and transfered to 6-well plates containing Iscove's medium (Sigma) with 2 mM glutamine and 20% heat-inactivated FCS. Analysis of specific silencing of PP4c, PP2cβ and PEA-15 expression was carried out after 48 h by qRT-PCR and Western blotting.

### 2.11 Real-time RT-PCR

The expression of PP4c was determined using real-time RT-PCR. 5 μg of RNA was reverse transcribed using SuperscriptTM II RNAse H-Reverse Transcriptase and random primers (Promega) according to the manufacturer's instructions (Invitrogen; #18064). Real-time PCR was performed using 2 μl of the cDNA (equivalent to 500 ng of the total RNA) with Taq Man MGB probes and primers specific to human PP4c (Applied Biosystems; Assay ID: Hs 00427262-m1) or human PP2cβ (Applied Biosystems; Assay ID: Hs Hs01038824_g1) with eukaryotic 18S rRNA as an endogenous control (Applied Biosystems; Assay ID: Hs99999901_s1), according to the manufacturer's instructions. Quantitation of PP4c or PP2cβ gene expression in PP4c- or PP2cβ-transfected clones and PP4c- and PP2cβ-knockdown cells, relative to cells transfected with empty vectors, or (−)siRNA cells, was determined using the comparative CT method, using parental cells as calibrators. The ABI Prism 7000 sequence detection system was used to measure real time fluorescence and data analysis was performed using ABI Prism 7000 SDS software.

### 2.12 Western blot analysis

Analysis of expression of PP4c (using anti-PP4c antibody; Santa Cruz #Sc6118; PPX/PP4 (C-18)) and PEA-15 (using anti-PEA-15 antibody; Santa Cruz Sc28255) was carried out as previously described [41].

### 2.13 Measurement of HPRT mutations

Radiation from the UVG-54 (UVP) lamp was routinely measured using the J-225 Black-Ray UV (shortwave) intensity meter (SER# 44725) and was 2 W/m^2^ at a distance of 25 cm. 10^6^ cells were exposed to UV in plastic Petri dishes with the lids removed for 20 s (40 J/m^2^). After UV exposure, the irradiated medium was replace by fresh complete RPMI medium and the cells were incubated for 10 days at 37 °C in 95% air:5% CO_2_, at an initial density of 5 × 10^5^ cells/ml, followed by cloning in soft agar in the presence (5 × 10^5^ cells/plate), or in the absence (200 cells/plate), of 50 μM 6-thioguanine (Sigma; #A4660). The mutation frequency was calculated as [(mean colony count per plate in presence of 6-thioguanine)/(cells per plate in selective medium)/surviving fraction in non-selective medium] [48].

### 2.14 Statistical analysis

Data are presented as the mean ± standard error of the mean (S.E.). Statistical significance was determined by analysis of variance using Origin 6.1. A *p*-value of <0.01 was considered statistically significant.

## 3 Results

### 3.1 Effects of PP4c over-expression on CEM-C7 and Jurkat T-cells

**Fig. 1:**
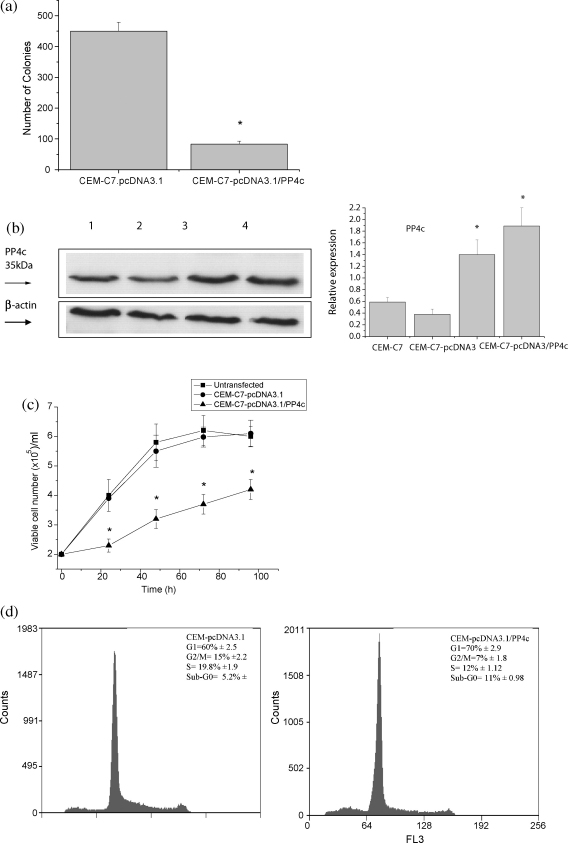
PP4c over-expression inhibits colony-forming ability, inhibits cell growth and increases apoptosis of CEM-C7 cells. CEM-C7 cells were transfected with either pcDNA3.1 or pcDNA3-PP4c. (a) 24 h post-transfection, cells were cloned in soft agar in the presence of G418 and the number of colonies was determined after 2–3 weeks. (b) Immunoblot of PP4c expression in CEM-C7 parental cells (lane 1), pcDNA3.1-transfected CEM-C7 cells (lane 2) and pcDNA.1-PP4c-transfected CEM-C7 cells (lanes 3 and 4). Each lane contains 50 μg of whole-cell lysate subjected to SDS-PAGE, followed by Western blot analysis with anti-PP4c antibody. Anti-β-actin antibody was used to reveal β-actin as a loading control. The resulting autoradiographs were analysed by densitometry. A representative autoradiograph is presented, and the bar graphs represent means ± S.E. from four independent experiments. Relative expression is the ratio of PP4c to β-actin. (c) Growth curve of CEM-C7, CEM-C7-pcDNA3.1-transcfected cells and CEM-C7-pcDNA3.1-PP4c-transfected cells over 96 h. Viable cell density was determined by nigrosin dye exclusion. Results are expressed as the means ± S.E., and are representative of data obtained from five separate experiments, *P < 0.01 compared with vector only and parental cells. (d) Cell cycle analysis of CEM-C7-pcDNA3.1-transfected cells and CEM-C7-pcDNA3.1-PP4c-transfected cells. DNA content was quantified by propidium iodide staining of fixed cells and fluorescence flow cytometry. Results are represented as the means ± S.E. (*n* = 5). Representative histograms are shown.

The EST clone PP4c (accession # BG913014) was cloned directionally in pcDNA3.1 and transfected into Jurkat and CEM-C7 cells to generate stable clones selected by growth in G418. Since very similar results were obtained from both cell lines, only CEM-C7 results are shown (the Jurkat cell data are provided as supplementary data online). Transfection of the PP4c expression construct into both cell lines led to the growth of 8- to 12-fold fewer colonies than vector-only transfected cells (Fig. 1a), reflecting the inhibitory effects of PP4c over-expression on cell growth and colony-forming ability. Three empty vector-containing clones and six PP4c-transfected clones from each cell line were further characterized. The degree of over-expression of PP4c was determined, firstly, by qRT-PCR (a 6–8-fold over-expression was observed (data not shown), and, secondly, by Western blotting (Fig. 1b). The effect of PP4c over-expression on CEM-C7 and Jurkat cell growth rate and apoptosis was examined. A significant difference in the rate of proliferation between cells transfected with PP4c and cells transfected with vector only was consistently observed. Fig. 1c shows that PP4c-transfected cells proliferated at a significantly lower rate than control pcDNA3.1-transfected cells. The observed regulatory effects of PP4c were not due to clonal idiosyncrasies, since the results were also confirmed using polyclonal populations of CEM-C7 and Jurkat cells transfected with PP4c or vector only (results not shown).

To determine whether the growth suppression by PP4c was due to increased apoptosis, to cell cycle arrest, or both, a cell cycle analysis was performed. The results revealed that the percentage of cells in G1 in transfected cells was considerably higher than that of the controls, suggesting that PP4c may induce arrest in G1 (Fig. 1d), with a corresponding decrease in cells in G2/M and S phases. In addition, PP4c-transfected clones showed a substantial increase in the sub-G1 fraction, indicating that the apoptosis rate in these clones is increased (Fig. 1d).

### 3.2 Effect of PP4c knockdown on apoptosis and cell proliferation

**Fig. 2:**
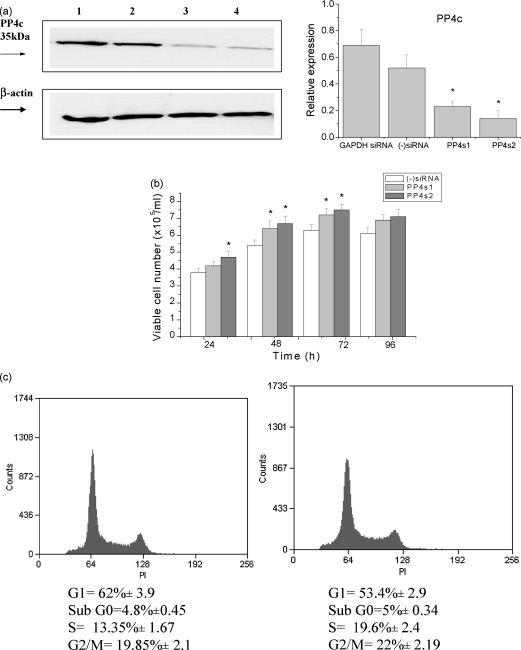
PP4c-specific siRNAs increase CEM-C7 proliferation and alter the cell cycle profile. CEM-C7 cells were transfected with control (−)siRNA or with PP4c-specific siRNA. (a) Expression of PP4c protein under the same conditions was determined by Western blotting and equivalent loading was demonstrated using anti-β-actin antibody. The resulting autoradiographs were analysed by densitometry. A representative autoradiograph is presented, and the bar graph shows means ± S.E. from five independent experiments. Relative expression is the ratio of PP4c to β-actin. (b) Viable cell number of (−)siRNA-, PP4s2- and PP4s1-siRNA-treated CEM-C7 cells over 96 h. *P < 0.01 compared with (−)siRNA. (c) Cell cycle profiles of (−)siRNA-transfected CEM-C7 cells and PP4s2 siRNA-transfected CEM-C7 cells. DNA content was quantified by propidium iodide staining of fixed cells and fluorescence flow cytometry. Results are represented as the means ± S.E. (*n* = 5). Representative histograms are shown.

In order to investigate PP4c function further in CEM-C7 and Jurkat cells, we used specific siRNAs, PP4cs1 and PP4cs2, to inhibit endogenous PP4c expression in these cells. This strategy is particularly important since the effects of over-expression of any protein should be confirmed by independent methods in order to exclude possible artefacts. The effects of PP4c siRNAs were similar in the CEM-C7 and Jurkat cell lines, therefore only CEM-C7 results will be shown here (results from Jurkat cell lines are provided as supplementary data). The efficiency of PP4c knockdown was determined by qRT-PCR and immunoblotting 48 h post-transfection and the specificity of the PP4c siRNAs was tested by comparison with GAPDH siRNA and the negative control siRNA ((−)siRNA). Both PP4c-targeted siRNAs (PP4cs1 and PP4cs2) reduced PP4c mRNA levels by more than 60%. PP4cs2 was more efficient in the silencing of PP4c (70% compared to 60%; data not shown), and this degree of silencing was maintained for up to 10 days (data not shown). The scrambled negative control and GAPDH siRNAs had no significant effect on PP4c mRNA levels. PP4c down-regulation was also confirmed by Western blot analysis (Fig. 2a). Following the knockdown of PP4c, cells were grown for 4 days and their proliferation rates were assessed based on viable cell counts. As shown in Fig. 2b, the growth rates of CEM-C7 cells transfected with PP4cs1 and PP4cs2 were significantly increased relative to (−)siRNA-transfected cells or GAPDH siRNA-transfected cells. PI staining and FACS analysis revealed increases in S and G2/M populations in the cells transfected with PP4cs1 and PP4cs2 siRNAs compared with the cells transfected with control (−)siRNA (Fig. 2c).

**Fig. 3:**
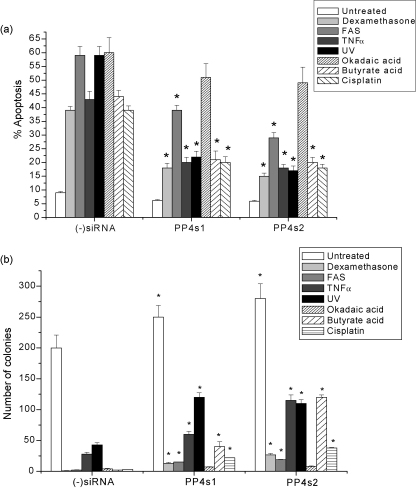
PP4c knockdown inhibits apoptosis induced by Dexamethasone, anti-Fas antibody, TNFα, UV, cisplatin and butyrate in CEM-C7 cells. 72 h post-siRNA transfection, (−)siRNA-, PP4s2- and PP4s1-siRNA-treated CEM-C7 cells were exposed to 10 μM dexamethasone, 5 ng/ml anti-Fas antibody, 50 ng/ml TNFα, 40 J/m^2^ UV, 5 μg/ml cisplatin, 5 mM butyrate or 30 nM okadaic acid. (a) Apoptosis was quantified after 48 h using CaspaTag staining. (b) Colony-forming assays were carried out at 72 h. Data represent means ± S.E. from five independent experiments, *P < 0.01 compared with (−)siRNA-transfected cells.

Since our original observation of the inhibition of apoptosis by PP4c down-regulation were made on the mouse thymoma cell line W7.2c challenged with dexamethasone and UV [40], we investigated the effects of PP4c down-regulation on Dexamethasone- and UV-induced apoptosis in CEM-C7 cells. Since induction of T-cell apoptosis through Fas/APO-1/CD95, TNFα, cisplatin, butyrate and okadaic acid has many features which are distinct from Dexamethasone- and UV-induced apoptosis, we also studied the effects of PP4c knockdown on CEM-C7 cell apoptosis induced by these stimuli. Cells were exposed to the apoptotic stimuli 48 h after transfection and apoptosis was quantified after 48 h using CaspaTag staining, which allows the detection of active caspases in live cells. Colony-forming assays, which allow the assessment of long-term survival and proliferation, were also carried out at 72 h. Fig. 3 shows that PP4c down-regulation significantly inhibited apoptosis induced by all the stimuli tested, except for okadaic acid, in both CEM-C7 (Fig. 3a) and Jurkat cells (see supplementary data) and also protected the colony-forming ability of these cells (Fig. 3b; supplementary data). The effect of PP4c knockdown on Dexamethasone-induced cell death in CEM-C7 cells was interesting. Although PP4c silencing significantly protected against Dexamethasone-induced apoptosis (Fig. 2a), the protective effects on colony-forming ability were relatively small (Fig. 3b). Down-regulation of PP4c did not protect against okadaic acid, most likely because okadaic acid has been reported to inhibit other phosphatases in addition to PP4 [21].

### 3.3 PP4c plays a critical role in apoptosis and cell proliferation in primary human T-lymphocytes

**Fig. 4:**
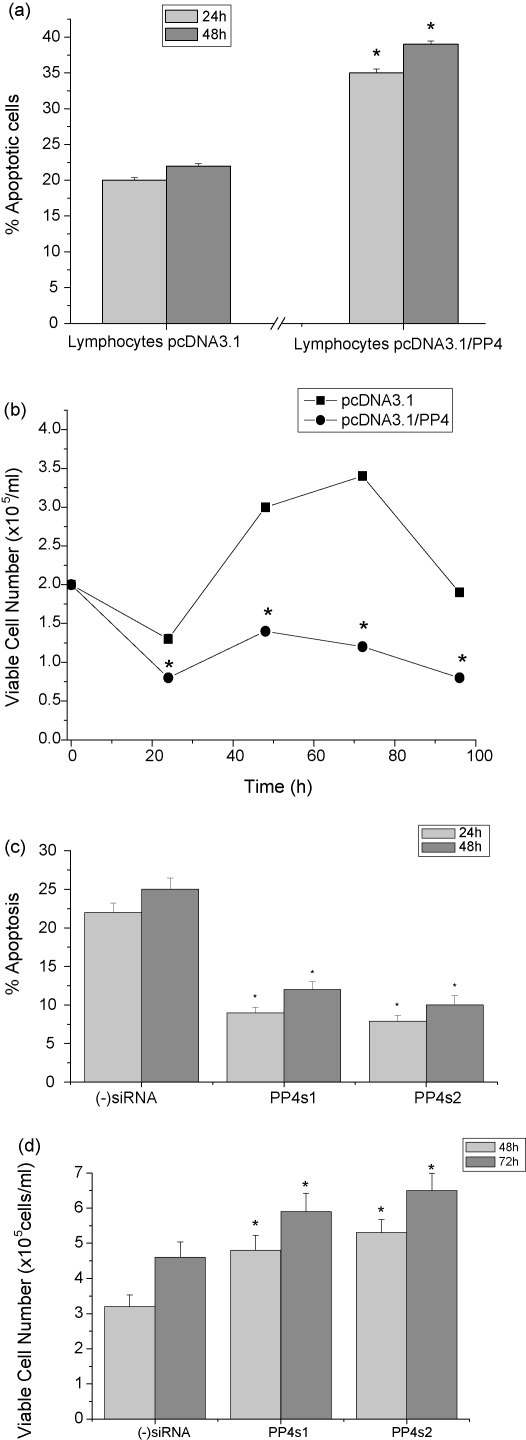
Up-regulation and down-regulation of PP4c expression produce complementary effects on the survival and growth of human peripheral blood lymphocytes. (Graphs (a) and (b)) Peripheral blood lymphocytes were cultured in complete RPMI medium supplemented with 2.5 μg/ml PHA for 5 days and transiently transfected with either pcDNA3.1 or pcDNA3.1-PP4c. (a) Apoptosis was determined at 24 and 48 h time points using CaspaTag (means ± S.E. from five independent experiments). (b) Viable cell numbers were determined by vital dye staining, at the indicated time points (means ± S.E. from seven independent experiments). (Graphs (c) and (d)) Peripheral blood lymphocytes were cultured in complete RPMI medium supplemented with 2.5 μg/ml PHA for 5 days and transfected with (−)siRNA, PP4s1 or PP4s2. (c) Caspase activation, as a marker of apoptosis, was determined at 24 and 48 h (means ± S.E. from six independent experiments). (d) Viable cell number was determined by vital dye staining (means ± S.E. from six independent experiments).

To confirm that PP4c expression plays a role in apoptosis and cell proliferation in untransformed lymphoid cells, we manipulated the endogenous levels of PP4c expression in human peripheral blood lymphocytes stimulated with the mitogen phytohaemagglutinin (PHA) [50]. PHA-stimulated T-lymphocytes were transiently transfected with pcDNA3.1-PP4c or with pcDNA3.1. Expression of PP4c was assessed by qRT-PCR 24 h post-transfection and was found to be increased by 4-fold (results not shown). Transfected cells were then plated for 4 days in the presence of PHA and their proliferation rate was monitored every 24 h, based on viable cell counting. Apoptosis was assessed after 24 and 48 h. PP4c-transfected cells showed a 1.5- to 2-fold increase in apoptosis relative to cells transfected with vector only (Fig. 4a), indicating that over-expression of PP4c by itself enhances apoptosis in normal human T-lymphocytes. Cells transfected with pcDNA3.1-PP4c showed a 3-fold decrease in the number of viable cells at every time point, compared to cells transfected with vector only (Fig. 4b), showing the inhibitory effect of PP4c on cell proliferation in primary human T-lymphocytes.

The effects of PP4c down-regulation on human peripheral blood lymphocytes were also investigated. PHA-stimulated cells were transfected with control (−)siRNA or PP4c-specific siRNAs, and PP4c expression was assessed 48 h post-transfection by qRT-PCR. The level of PP4c in the cells transfected with PP4c siRNAs was decreased by 60–70% in comparison to the cells transfected with (−)siRNA (results not shown). Fig. 4c shows that PP4c knockdown significantly protected T-lymphocytes from spontaneous apoptosis, reducing the level of apoptosis to less than half that in control cell populations transfected with (−)siRNAs. In addition, the number of viable cells in cultures transfected with PP4c siRNAs was significantly higher at both 24 and 48 h than the number of viable cells in cultures transfected with (−)siRNAs. This is due to both inhibition of apoptosis and stimulation of the cell proliferation rate (Fig. 4d). The increase in T-lymphocyte proliferation caused by PP4c down-regulation was confirmed by Ki67 labelling of proliferating cells [51] (supplementary data).

**Fig. 5:**
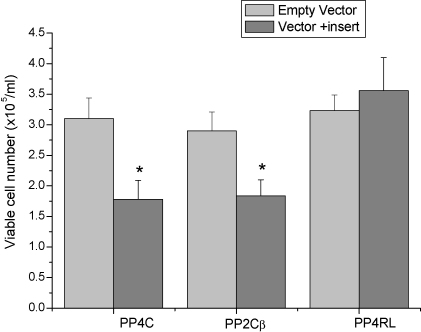
Comparison between the effects of PP2cβ, PP4 phosphatase-dead mutant PP4-RL, and PP4c on viable cell number of human peripheral blood lymphocytes. PHA-stimulated lymphocytes were transfected with pcDNA3.1-PP4c or pcDNA3.1, pCMVSPORT6-PP2c or pCMVSPORT, or the phosphatase-dead PP4c mutant in the expression vector expression vector pCI-neo. Viable cell number was determined after 48 h. Results represent means ± S.E. and are representative of data obtained from five independent experiments, *P < 0.01 compared with vector only.

Further studies were carried out to compare the effects of PP4c with those of PP2cβ (protein phosphatase 2 catalytic subunit β isoform), which has previously been reported to be associated with cell death [52]. We have also used a functionally deficient PP4c mutant [32,38] in order to ensure that the effects produced by PP4c up-regulation were due to the catalytic activity of PP4c. PHA-stimulated lymphocytes were transfected with pcDNA3.1-PP4c or pcDNA3.1, pCMVSPORT6-PP2cβ (accession # BC012022) or pCMVSPORT and the phosphatase-dead PP4c mutant in the expression vector pCI-neo (Promega) (pCI-PP4-RL), in which arginine (Arg-235) in the PP4c phosphatase domain has been replaced by leucine [32,38]. The expression of PP4c and PP2cβ was assessed by RT-PCR 24 h post-transfection and was found to be increased by 4.4-fold on average for PP4c and 5-fold for PP2cβ, as compared to cells transfected with empty vector. Over-expression of PP2cβ caused a decrease in viable cell number which was of a similar magnitude to that produced by PP4c, as assessed by cell counting 48 h post-transfection. Transfection with the non-functional mutant of PP4c, PP4-RL, did not have the same effect as the two catalytic subunits of PP4 and PP2, since the number of viable cells from these cultures was actually slightly higher than the number of viable cells from cultures transfected with empty vector, although the increase in cell number was not statistically significant (Fig. 5).

**Fig. 6:**
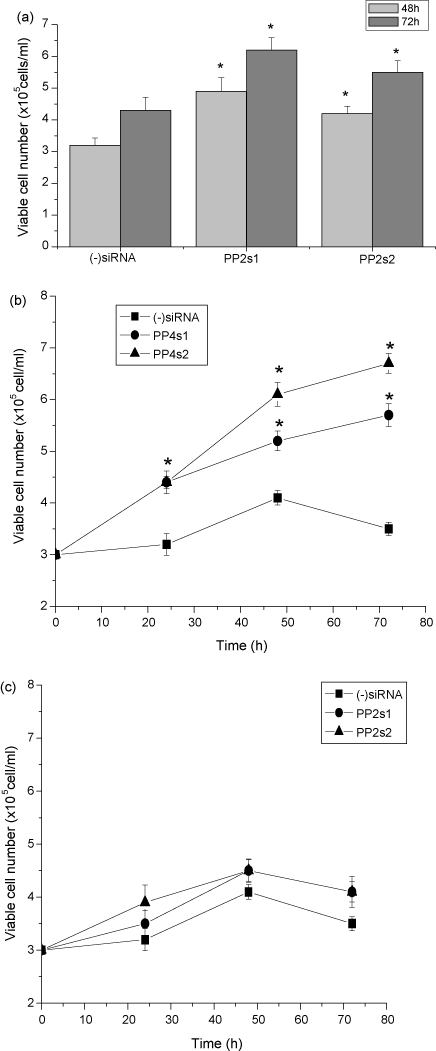
Effects of PP2cβ down-regulation on PHA-stimulated human peripheral blood lymphocyte cell viability. PHA-stimulated human peripheral blood lymphocytes were transfected with either (−)siRNA or one of the two specific PP2cβ-targeted siRNAs. (a) Viable cell number was determined after 48 and 72 h. Results represent means ± S.E. and are representative of data obtained from five independent experiments, *P < 0.01 compared with (−)siRNA. (b) and (c) Comparison between the effects of PP4c (b) and PP2cβ (c) down-regulation on growth inhibition of human peripheral blood lymphocytes induced by fetal calf serum withdrawal. Cell density was determined by nigrosin dye exclusion at different time points. Results are expressed as the means ± S.E., and are representative of data obtained from five separate experiments, *P < 0.01 compared with (−)siRNA transfected cells.

The effects of PP2cβ down-regulation on PHA-stimulated human peripheral blood lymphocytes, using two specific PP2cβ-targeted siRNAs, were also investigated and compared to those of PP4c down-regulation. Transfection of PP2cβ siRNAs resulted in an average 70% decrease in the endogenous level of PP2cβ when using PP2s1 and in a 60% decrease using PP2s2, as assessed using real time RT-PCR. Fig. 6a shows that down regulation of PP2cβ resulted in an increase in cell survival and proliferation. As compared to negative control siRNA, transfection with either of the two PP2cβ siRNAs produced a significant increase in viable cell number both at 48 and 72 h (Fig. 6a). The effects of PP2cβ down-regulation on viable cell number were similar to those produced by the knockdown of PP4c (Fig. 4b). However, a remarkable difference between the effects of down-regulation of the two phosphatases was revealed when the transfected cells were incubated in the absence of fetal calf serum. Cells transfected with PP4c siRNAs were more resistant to serum withdrawal, relative to cells transfected with control siRNA (Fig. 6b), while the growth of cells transfected with PP2cβ siRNAs was similar to those transfected with control siRNA (Fig. 6c).

### 3.4 The effects of PP4c expression on T-cell survival and mutation after UV irradiation

**Fig. 7:**
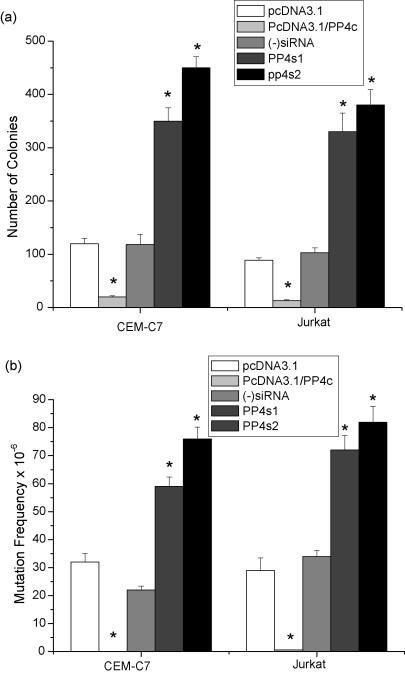
Modulation of PP4c expression affects colony-forming ability and mutation frequency at the HPRT locus in CEM-C7 and Jurkat cells. (a) CEM-C7 and Jurkat cells transfected with either pcDNA3.1, pcDNA3.1-PP4c, (−)siRNA, PP4s1 or PP4s2 siRNAs were exposed to 40 J/m^2^ UV, and colony-forming ability was determined 48 h after UV exposure. Results are expressed as means ± S.E. from five independent experiments, *P < 0.01 compared with vector-only and parental cells. (b) Mutation frequencies at the HPRT locus in CEM-C7 and Jurkat cells transfected with either pcDNA3.1, pcDNA3.1-PP4c, (−)siRNA, PP4s1 or PP4s2 were determined 10 days post-UV irradiation by cloning in soft agar in the presence or absence of 50 μM 6-thioguanine. Data represent means ± S.E. from five independent experiments. *P < 0.01 compared with vector only and (−)siRNA transfected cells.

Over-expression of PP4c has been shown to decrease the mutation rate in the UV-treated embryonic kidney cell line HEK 293T [41]. We therefore examined the effects of PP4c on cell survival and determined the frequency of mutation at the hypoxanthine-guanine phosphoribosyl transferase (HPRT) locus after UV irradiation of human leukemic T-cell lines. We compared HPRT mutation frequencies between control cells and cells transfected with pcDNA3-PP4c or PP4c siRNAs. The colony-forming ability of CEM-C7 and Jurkat cells was substantially reduced after UV exposure 40 J/m^2^ (results not shown). pcDNA3.1-PP4c transfection enhanced the loss of colony-forming ability of these cells (Fig. 7a). On the other hand, suppression of PP4c significantly protected from the loss of colony forming ability induced by UV (Fig. 7a). We therefore analysed the production of mutant HPRT cells after UV exposure in pcDNA3.1-PP4c-transfected cells and PP4c siRNA-transfected cells. A significant decrease in the mutation rate was seen in PP4c-transfected cells (Fig. 7b), whereas cells transfected with PP4c siRNAs showed a corresponding substantial increase in the mutation rate (Fig. 7b). Taken together, these data further indicate an important role for PP4c in the apoptosis which normally acts to eliminate mutated cells.

### 3.5 Interaction between PP4c and PEA-15

**Fig. 8:**
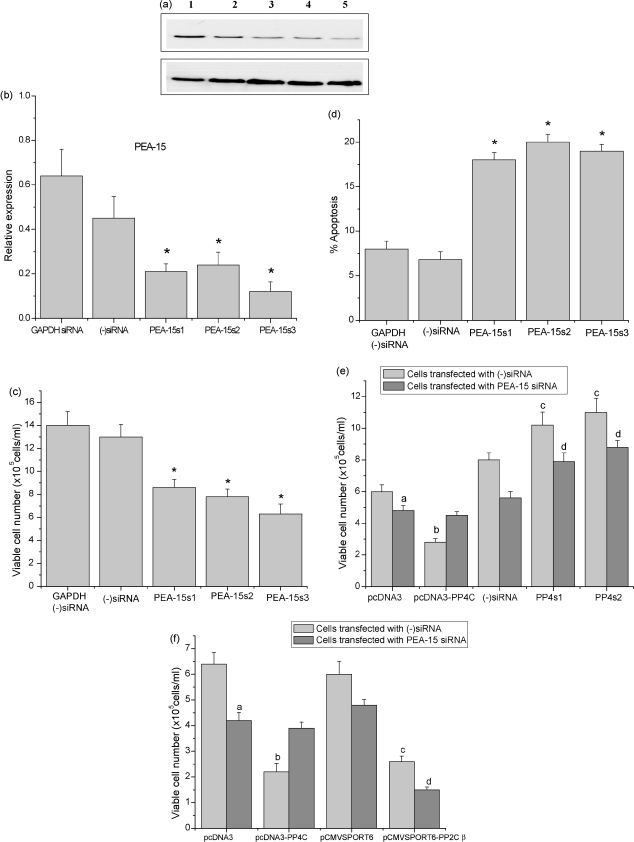
PEA-15-specific siRNAs inhibit cell growth and increase apoptosis in CEM-C7 cells. CEM-C7 cells were transfected with control (−)siRNA, GAPDH siRNA or with three different PEA-15-specific siRNAs. (a) Expression of PEA-15 protein under the same conditions was determined by Western blotting and equivalent loading was demonstrated using anti-β-actin antibody. The resulting autoradiographs were analysed by densitometry. A representative autoradiograph is presented, and the bar graph (b) shows the means ± S.E. from five independent experiments. Relative expression is the ratio of PEA-15 to β-actin. (c) Viable cell count of CEM-C7 cells treated with GAPDH siRNA, (−)siRNA or PEA-15s1, PEA-15s2 or PEA-15s3 siRNA, after 48 h. Data represent means ± S.E. from five independent experiments, *P < 0.01 compared with (−)siRNA transfected cells. (d) Active caspase staining, as a marker of apoptosis, was determined using CaspaTag and fluorescence microscopy. Data shown are the means ± S.E from five separate experiments, *P < 0.01 compared with (−)siRNA-transfected cells. (e) Cell density was determined by nigrosin dye exclusion. (f) Effects of PP2cβ over-expression on PEA-15 siRNA-transfected cells. CEM-C7 cells were transfected with either control (−)siRNA or PEA-15-specific siRNA. 48 h post-transfection, control cells (transfected with negative control siRNA) and cells transfected with PEA-15 siRNAs were transiently transfected with pCMVSPORT6-PP2cβ or pCMVSPORT6. Viable cell number was determined after 24 h. Data represent means ± S.E. from five independent experiments. (a) *P* < 0.01 compared with (−)siRNA. (b) *P* < 0.01 compared to pcDNA3.1. (c) *P* < 0.01 compared to (−)siRNA. (d) *P* < 0.01 compared to pCMVSPORT6.

Phosphoprotein enriched in astrocytes 15 kD (PEA-15) is one of the proteins that appears to be regulated, directly or indirectly, through dephosphorylation by PP4c [41]. In order to investigate whether PEA-15 plays a major role in mediating the apoptosis-inducing effects of PP4c, we used PEA-15-specific siRNAs to down-regulate PEA-15 and then studied the effects of modulation of PP4c expression on the viability of these cells. CEM-C7 cells were transfected with PEA-15-specific siRNAs. 48 h post-transfection, PEA-15 down-regulation was assessed by real-time RT-PCR and Western blotting (Fig. 8a and data not shown), and cell viability was determined. All three siRNAs were able to down-regulate PEA-15 expression to a similar extent (60–70%; Fig. 8a and data not shown). Down-regulation of PEA-15 caused a decrease in the number of viable cells and more than doubled the proportion of apoptotic cells, confirming the involvement of PEA-15 in regulating leukemic cell proliferation and apoptosis (Fig. 8c and d). 48 h post-transfection, control cells (transfected with negative control siRNA) and cells transfected with PEA-15 siRNAs were transiently transfected with pcDNA3.1-PP4c, pcDNA3.1 (−)siRNA or PP4c-specific siRNAs (transfection efficiency 50–60%). As similar results were obtained from all three PEA-15 siRNAs, data obtained using only one PEA-15 siRNA are shown. In the cells transfected with (−)siRNA, over-expression of PP4c caused 50% reduction in viable cell number, confirming the results obtained earlier (Fig. 8e), whereas down-regulation of PP4c caused a 50–60% increase in viable cell number (Fig. 8e). On the other hand, cells transfected with PEA-15 siRNAs showed a decrease in viable cell number in control cells (cells transfected with (−)siRNA and vector only) (Fig. 8e). Over-expression of PP4c in these cells did not have any additional effect on cell viability as it was found that the viable cell number of (−)siRNA transfected cells was similar to that of PP4c transfected cells (Fig. 8e). However, PP4c-specific siRNAs partially reversed the effects of PEA-15 down-regulation (Fig. 8e). In order to ensure that the effects of PEA-15 down-regulation on PP4c function were specific, we analysed the effects of PP2cβ over-expression on PEA-15 siRNA transfected cells. Cells transfected with (−)siRNA or PEA-15 siRNAs were transiently transfected with pCMVSPORT6-PP2cβ or pCMVSPORT6, and viable cell number was determined after 24 h. Fig. 8f shows that, in contrast to PP4c, transfection of PEA-15 knock-down cells with PP2cβ led to an additional loss of viable cells, confirming that although PP4c and PP2cβ have similar effects on cell viability, their mechanisms of action are different.

## 4 Discussion

Here we report that PP4c exerts profound and specific effects on the growth and survival of both normal and leukemic human T-cells. The data presented reveal that over-expression of PP4c in the T-leukemic cell lines CEM-C7 and Jurkat inhibited their proliferation in the absence of extracellular apoptotic stimuli by inducing apoptosis and cell cycle arrest in G1. Our work also showed that down-regulation of endogenous PP4c by 60–70% increased the rate of cell proliferation and conferred resistance to a number of apoptotic stimuli. While down-regulation of PP4c conferred substantial resistance to UV, cisplatin and butyrate, it protected against Fas-induced apoptosis only partially. This partial inhibition of Fas-induced apoptosis caused by down-regulation of PP4 may be explained in part by recognizing that, in addition to caspase-dependent pathways, Fas ligation activates other major signaling cascades that belong to the family of mitogen-activated protein kinase (MAPK) pathways [52,53]. Down-regulation of PP4c protected against Dexamethasone-induced apoptosis but was less effective in protecting against the loss of colony-forming ability. This may indicate that PP4c down-regulation protects only against dexamethasone-induced apoptosis and is less effective against the anti-proliferative effects of dexamethasone. These results suggest that PP4 may be able to modulate apoptosis in leukemic T-cells via a common factor which affects the apoptosis signaling pathways mediated by these different apoptotic stimuli. The modulation of PP4c level did not affect cell death induced by okadaic acid, presumably because okadaic acid also inhibits other phosphatases, particularly PP2, with a comparable IC_50_ [55]. The critical importance of PP4c levels for the growth and survival of T-cells was further demonstrated using primary cultures of PHA-stimulated human peripheral blood lymphocytes. As for the leukemic T-cell populations, our data show that over-expression of PP4c caused inhibition of normal lymphocyte proliferation and an increase in apoptosis, whereas down-regulation of PP4c was accompanied by a corresponding increase in proliferation and inhibition of apoptosis. Our results do not conflict with previous work which showed that PP4c activity is required for microtubule nucleation during centrosome maturation in *Caenorhabditis elegans* [29], and for the development of thymocytes in mice and to have an anti-apoptotic role in thymocytes [42], since these observations can quickly be reconciled by recognition of the wide range of cellular processes which involve PP4c. It is therefore likely that a minimum level of this enzyme is required for survival and proliferation. Since our analysis was inevitably focused on viable cells capable of replication and colony formation, we would not have detected any cells where the PP4 activity was below the minimum required for centrosome maturation.

Recent studies have shown that PP4 regulates an increasing number of cellular functions in different cellular locations and have resulted in the identification of several PP4 regulatory subunits and binding proteins [22,24,29,31]. Like other serine/threonine phosphatases, PP4 is probably targeted to its specific sites of action by these regulatory subunits and it is likely that the interchange between the different regulatory subunits and binding proteins plays a critical role in regulating the activity of PP4 complexes. It is also important to note that PP4c is a positive regulator of HPK1 [38], which in turn is known to promote apoptosis of murine T lymphocytes [57,58]. The possibility that over-expression of PP4c promotes apoptosis via the activation of HPK1 therefore requires further investigation.

It is also believed that loss of DNA repair systems that prevent the fixation of premutagenic lesions in the genome is mandatory for carcinogenesis, since the increased mutation rates of unstable cells can give them a growth advantage over normal cells and allow them to accumulate aberrations that subsequently lead to cancer [59]. In the present study, we monitored the rate of mutation at the HPRT marker locus in order to study the effects of PP4c modulation on the mutation rate of leukemic T-cells. In agreement with our previous studies using HEK 293T cells [41], our results showed that over-expression of PP4c significantly reduces mutation at the HPRT locus. These effects were dependent on PP4c catalytic activity since the PP4c phosphatase-dead mutant (PP4-RL) did not affect the mutation rate of these cells (results not shown). On the other hand, down regulation of endogenous PP4c consistently increased the HPRT gene mutation frequency. These observations have important implications for oncogenesis and further support an important rate-limiting role for PP4c in the induction of apoptosis after DNA damage.

PP4 shares 65% amino acid identity with PP2, the most abundant serine/threonine phosphatase in mammals [23,24], which plays a crucial role in many fundamental processes including differentiation, embryonic development, and growth control [60,61]. The great versatility of PP2 is a result of the existence of a large number of subunits: two isoforms of the catalytic C subunit (Cα and Cβ), two isoforms of the regulatory/scaffolding A subunit (Aα and Aβ) and numerous regulatory B subunits that fall into four families designated B, B′, B″, and ${\text{B}}^{\prime \prime \prime }$ [62–66]. In addition, PP2, PP4 and PP6 share the alpha 4 (α4) protein, the mammalian ortholog of yeast Tap42, which binds to the catalytic subunits and displaces other regulatory subunits [28,67]. Several lines of evidence implicate PP2 in the regulation of apoptosis [28,43,44,61,68]. However, as for PP4, the reported effects of PP2 on apoptosis and cell growth appear variable, since its function has been variously described as pro-apoptotic [69,70] and as anti-apoptotic [71]. Functional screens of human phosphatases have identified the α isoform of PP2 catalytic subunit as associated with survival and the β isoform as associated with cell death [51]. Our data indicate that PP2cβ over-expression causes an inhibitory effect on cell proliferation and enhances apoptosis, producing effects similar to those observed with PP4c in these cells. In addition, as with the knockdown of PP4c, down-regulation of PP2cβ increased cell proliferation. However, while PP4c knock-down conferred resistance to fetal calf serum withdrawal, reducing PP2cβ levels had little effect in this situation, indicating that each of these phosphatases acts independently on different apoptosis pathways. α4 protein, a PP2- and PP4-regulatory subunit, has been implicated in the regulation of B- and T-cell differentiation, embryonic development and cell death [71–73]. Knockout of α4 decreases cell proliferation and promotes apoptosis in thymocytes as well as in other cell types [74,75]. α4 has been shown to interact directly with PP6, PP4c and PP2 catalytic subunits and exerts opposing kinetic effects on these target phosphatases [28]. It is likely that the pro-apoptotic effects observed as a result of α4 knockdown are produced by the increased activity of PP4c and/or the inhibition or activation of PP2 catalytic subunits. Further work is required to investigate the interplay between PP4c, PP2 catalytic subunit isoforms and α4 and to determine their roles in commitment to apoptosis.

A multiplexed phosphorylation screen revealed that modulation of the endogenous level of PP4c in HEK 293T cells resulted in an increase or reduction in the phosphorylation state of many proteins involved in cellular stress, cellular proliferation and apoptosis [41]. The phosphorylation of PEA-15 was found to increase dramatically (by 223%) when PP4c expression was reduced [41]. PEA-15 has been reported to modulate signalling pathways that control apoptosis and cell proliferation [45,46] and to play a rate-limiting role in the induction of B-CLL cell apoptosis induced by TRAIL [47]. PEA-15 can act to modulate apoptosis and as a critical regulator of the cell cycle. Its function is tightly regulated by phosphorylation and is involved in the signalling pathways mediated by ERK1/2, Akt and RSK2 [45,46,56,76,77]. In the present study, we have investigated the involvement of PEA-15 in mediating the effects of PP4c on T-leukemic cells. Down-regulation of PEA-15 reduced viable cell number, in accordance with its reported effects as an anti-apoptotic protein. PP4c over-expression in the cells which have down-regulated PEA-15 did not cause additional cell death, in contrast to the effects of PP2cβ over-expression. These observations suggest that the induction of apoptosis by over-expression of PP4c is mediated, at least in part, by the dephosphorylation of PEA-15. This highlights clear differences between the pathways which mediate the effects of PP4c and those that mediate the effects of PP2, which appear to be largely independent of PEA-15. Down-regulation of PP4c was able to partially reverse the inhibitory effects of PEA-15 knockdown, suggesting that PP4c activity is normally an important element in regulating PEA-15 activity. The interaction between PEA-15 and PP4c may therefore be critical in leukemogenesis and/or leukemia progression. The PEA-15 gene is amplified in breast cancer as well as in other cancers [78], where PEA-15 over-expression confers resistance to abroad range of anti-cancer drugs [79]. Since PEA-15 also confers TRAIL-resistance on leukemic cells [47], and the reistance conferred depends on the phosphorylation status of PEA-15 [54], PP4 activity is likely to play an important role in regulating leukemic cell drug sensitivity. Akt, which phosphorylates and stabilises the anti-apoptotic action of PEA-15, is also upregulated in a number of human cancers [80], suggesting that they might function cooperatively in tumorigenesis. Developing strategies that enhance the activity of PP4c to oppose the effects Akt on PEA-15 could therefore prove to be effective in treating cancer.

## 5 Conclusions

It is clear that multiple cellular functions are regulated by PP4c and its interacting proteins and that several of these are crucial to determining leukemic cell survival, particularly after treatment with cytotoxic drugs. Our findings indicate that the endogenous level of PP4c plays a critical role in maintaining the delicate balance between cell survival and cell death both in normal lymphocytes and T-leukemic cells and acts, at least partly, through direct or indirect dephosphorylation of PEA-15. These observations suggest that modulating PP4c or PEA-15 function may prove important in therapeutic strategies for the treatment of leukemia.
