# Supplementary figures and images for: Protein phosphatase 4 regulates apoptosis in leukemic and primary human T-cells
Source: Leuk Res. 2009 Nov;33(11):1539–51. doi: 10.1016/j.leukres.2009.05.013 (PMC2734887; doi:10.1016/j.leukres.2009.05.013)

Supplementary Figure 1e


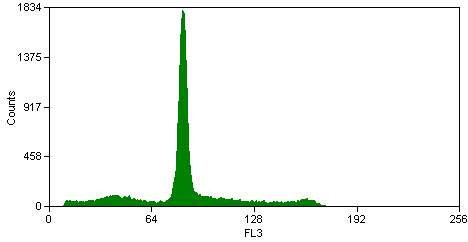


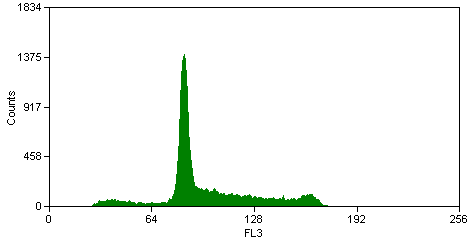


Jurkat-pcDNA3.1

G1=57% ± 3.2

G2/M= 18% ±2.2

S=18.7% ±1.9

Sub-G0= 6.3% ± 0.32

Jurkat-pcDNA3.1/PP4c

G1=69% ± 1.8

G2/M= 8% ± 1.1

S= 11% ± 0.97

Sub-G0= 12% ± 0.68

Supplement: Supplementary file 5 [file mmc5.doc]
