# Supplementary material for: Protein phosphatase 4 regulates apoptosis in leukemic and primary human T-cells
Source: Leuk Res. 2009 Nov;33(11):1539–51. doi: 10.1016/j.leukres.2009.05.013 (PMC2734887; doi:10.1016/j.leukres.2009.05.013)
Supplement: Supplementary file 11 [file mmc11.doc]

Supplementary Figure 4b
