# Supplementary material for: Protein phosphatase 4 regulates apoptosis in leukemic and primary human T-cells
Source: Leuk Res. 2009 Nov;33(11):1539–51. doi: 10.1016/j.leukres.2009.05.013 (PMC2734887; doi:10.1016/j.leukres.2009.05.013)
Supplement: Supplementary file 14 [file mmc14.doc]

**Legends to Supplementary Figures**

Supplementary Fig. 1. PP4c overexpression inhibits colony-forming ability of Jurkat cells and inhibits cell growth and increases apoptosis. Jurkat cells were transfected with either pcDNA3.1 or pcDNA3-PP4c. (a) 24h post transfection, cells were cloned in soft agar in the presence of G418 and the numbers of colonies were determined after 2-3 weeks. (b) The expression of PP4c in Jurkat cells transfected with pcDNA3/PP4c relative to cells transfected with pcDNA3.1 was determined by qRT-PCR. Results are represented as mean ± S.E. from four separate experiments, * P<0.01 compared with vector only. (c) Growth curve of Jurkat, Jurkat-pcDNA3.1-transcfected cells and Jurkat- pcDNA3.1/PP4c-transfected cells over 96 h. Cell density was determined by nigrosin dye exclusion. Results are expressed as the means ± S.E., and are representative of data obtained from five separate experiments, * P<0.01 compared with vector only and parental cells. (d) Active caspase staining, as a marker of apoptosis, was determined using CaspaTag kit and fluorescence microscopy. Data are represented as the means ± S.E from five separate experiments, * P<0.01 compared with vector only. (e) Cell cycle analysis of Jurkat-pcDNA3.1-transfected cells and Jurkat-pcDNA3.1/PP4c-transfected cells. DNA content was quantified by propidium iodide staining of fixed cells and fluorescence flow cytometry. Results are represented as the means ± S.E. (n=5). Representative histograms are shown.

Supplementary Fig. 2. Knockdown of PP4c expression level in Jurkat cells using PP4c-specific siRNA increases their proliferation. Jurkat cells were transfected with control (-)siRNA or with PP4c-specific siRNAs. (a) 48h after transfection, the expression of endogenous PP4c RNA was determined by qRT-PCR. The expression of endogenous PP4c in Jurkat cells transfected with PP4c siRNAs was compared with that in the cells transfected with (-) siRNA. Results are represented as means ± S.E. (for PP4c expression relative to untransfected cells) from five separate experiments, * P<0.01 compared with (-)siRNA. (b) Viable cell number of (-)siRNA-, PP4s2- and PP4s1-siRNA-treated Jurkat cells over 96 h. * P<0.01 compared with (-)siRNA.

Supplementary Fig. 3. Down regulation of PP4c inhibits apoptosis induced by a number of apoptotic stimuli in Jurkat T-cells. 72h post siRNAs transfection, (-)siRNA- and PP4s2 and PP4s1 siRNA-transfected Jurkat cells were exposed to 5ng/ml anti-Fas antibody, 40J/m^2^ UV, 5μg/ml cisplatin, 5mM butyrate or 30nM okadaic acid. (a) Apoptosis was quantified after 48h using CaspaTag staining. (b) Colony-forming assays were carried out at 72h.. Data represent means ± S.E. from five independent experiments, * P<0.01 compared with (-)siRNA-transfected cells. Note that Jurkat JKM1 cells are not sensitive to TNFα, so were not challenged with it.

Supplementary Fig. 4. Histone deacetylase activity is regulated by PP4c. Histone deacetylase activity (HDAC) was determined in extracts from CEM-C7 and Jurkat cells transfected with either pcDNA3.1, pcDNA3.1/PP4c(a), (-)siRNA, PP4s1 or PP4s2 siRNAs (b), as described in Materials and Methods, following the manufacturer’s instructions. HDAC activity was expressed as the relative OD value per μg protein sample. Representative results for one of three experiments performed is shown, mean ± S.E., n=5. * P<0.01 compared to the relevant control.

Supplementary Figure 5. PP4c-specific siRNAs protect against apoptosis and regulate the growth of human peripheral blood lymphocytes. Peripheral blood lymphocytes were cultured in complete RPMI medium supplemented with 2.5μg/ml PHA for 5 days and transfected with (-)siRNA, PP4s1 or PP4s2. (A) Percentage of Ki67 positive cells. Data represent means ± S.E. from five independent experiments, * P<0.01 compared with (-)siRNA transfected cells. Data represent means ± S.E. from five independent experiments. (B) Representative photograph of human peripheral blood lymphocytes (-)siRNA , PP4s2 and PP4s1 labelled with Ki67.
